# Incomplete TNM Documentation in Gastric Cancer: Frequency, Phenotype, and Treatment Allocation

**DOI:** 10.3390/diagnostics16060870

**Published:** 2026-03-15

**Authors:** Alexandru-Marian Vieru, Maria-Lorena Mustață, Virginia-Maria Rădulescu, Emil Trașcă, Sergiu-Marian Cazacu, Petrică Popa, Tudorel Ciurea

**Affiliations:** 1 Doctoral School, University of Medicine and Pharmacy of Craiova, 200349 Craiova, Romania; alexandruvieru1993@gmail.com; 2 Faculty of Medicine, University of Medicine and Pharmacy of Craiova, 200349 Craiova, Romania; etrasca@yahoo.com (E.T.); sergiu.cazacu@umfcv.ro (S.-M.C.); popa.petrica@umfcv.ro (P.P.); tudorel.ciurea@umfcv.ro (T.C.)

**Keywords:** gastric cancer, real-world evidence, TNM staging, metastatic disease, treatment allocation, tumor markers, phenotypes

## Abstract

**Background/Objectives:** Real-world gastric cancer cohorts often show incomplete TNM documentation, which can affect the interpretation of stage, phenotype, and treatment allocation. We aimed to quantify staging completeness, describe advanced-disease phenotype, and examine treatment selection at diagnosis in a real-world gastric cancer cohort. **Methods:** We performed a retrospective observational study of consecutive patients diagnosed with gastric cancer at a tertiary referral center. Data included age, sex, TNM components, metastatic status, surgery (any vs. none), and available serum markers (CEA, CA19-9). Incomplete staging was defined a priori as Tx and/or Nx and/or Mx. The primary endpoint was metastatic disease at diagnosis (M1) among patients with defined M status. In TNM-complete cases, a composite locally advanced or metastatic endpoint (LAM: M1 or T4 or N2–N3) supported sensitivity analyses. Logistic regression assessed associations with M1 and treatment allocation without biomarker cut-offs (markers modeled as continuous covariates). **Results:** The cohort included 419 patients. Incomplete staging was observed in 36.8%. M status was defined in 89.5%, with M1 in 52.0% of M-defined cases. Surgery was less frequent in M1 than M0 patients (34.4% vs. 73.3%; *p* < 0.001). Phenotype stratification showed a marked difference in surgical allocation, which was highest in M0-LAM (89.1%) and lowest in M1 (48.4%). Marker associations were directionally coherent but not definitive. **Conclusions:** Incomplete staging is common and clinically relevant in real-world gastric cancer and should be reported explicitly. Phenotype-based summaries provide a pragmatic framework for interpreting advanced disease and treatment selection, while tumor markers should be interpreted cautiously without predefined cut-offs.

## 1. Introduction

Gastric cancer remains a substantial global health burden and is still among the leading causes of cancer-related death worldwide, even though incidence has declined in several regions over the past decades [1,2,3,4,5]. Outcomes are closely tied to the stage at diagnosis, and in everyday practice many patients continue to present with advanced or metastatic disease [6,7,8,9]. For this reason, staging at diagnosis is not simply a reporting exercise. It directly shapes prognostic counselling, treatment selection, and the comparability of real-world cohorts.

Current guidelines place comprehensive TNM staging at the center of initial assessment, relying on depth of invasion, nodal involvement, and the presence of distant metastases to guide management [10,11,12,13,14]. In practice, however, diagnostic pathways rarely match the clean conditions assumed in guidelines or trials. Work-up intensity varies with referral routes, clinical urgency, access to imaging, and local documentation habits. It is therefore common to encounter partially documented TNM components in institutional datasets and registries, with Tx, Nx, and or Mx recorded when information is unavailable or not captured [15,16,17].

Many observational studies either exclude such cases or treat incomplete TNM data as a form of non-differential missingness. Both approaches can be problematic. Exclusion risks selecting a subgroup with systematically different diagnostic trajectories and disease severity, while assumptions about the “likely” values of missing TNM components can shift apparent stage distributions and distort downstream comparisons [15,18]. Although this issue has been raised in registry-based research, real-world gastric cancer studies still infrequently report staging completeness explicitly or examine how incomplete documentation relates to advanced presentation and treatment allocation.

A second, related gap concerns how real-world cohorts are summarized at diagnosis. Analyses often rely on broad stage groupings or focus primarily on survival endpoints, yet clinicians typically make early decisions by separating non-metastatic disease that is potentially resectable from locally advanced disease and from overt metastatic disease. A reproducible, phenotype-oriented stratification that mirrors these decision branches may therefore be more informative when describing routine care pathways and treatment patterns [19,20]. Serum tumor markers such as carcinoembryonic antigen and carbohydrate antigen 19-9 are also frequently recorded in clinical practice, but their contribution to phenotype characterization in real-world datasets is less clear, particularly when modeled continuously rather than forced into arbitrary cut-offs [21,22,23,24,25].

Beyond anatomical staging, growing evidence indicates that biological and microenvironmental factors, including alterations in the gut microbiome, may also contribute to gastric carcinogenesis and disease progression [26].

In this context, we aimed to describe staging completeness at diagnosis, characterize advanced-disease phenotypes, and examine initial treatment allocation in a consecutive, real-world gastric cancer cohort. We prespecified incomplete staging as Tx and/or Nx and/or Mx, used metastatic disease at diagnosis (M1) as the primary endpoint among patients with documented M status, and applied a phenotype-based framework in TNM-complete cases to support sensitivity analyses. Our goal was to provide a transparent, reproducible account of real-world diagnostic and treatment pathways that can be interpreted alongside guideline-based and trial-derived evidence.

## 2. Materials and Methods

### 2.1. Study Design and Setting

This retrospective observational study was conducted at the Oncology and Surgical Departments of the Craiova County Emergency Hospital, a tertiary referral center in southern Romania. The study included consecutive patients newly diagnosed with gastric cancer between January 2018 and December 2021. The analysis was performed using data prospectively collected in the institutional oncology registry and supplemented by chart review for missing variables. The study aimed to compare the clinical profile, disease stage, treatment allocation, and survival outcomes between the pre-pandemic period (2018–2019) and the pandemic period (2020–2021).

### 2.2. Study Population and Operational Definitions

#### 2.2.1. Eligibility Criteria

Eligible participants were adult patients with a new diagnosis of gastric cancer documented within the study window and managed through institutional clinical pathways. Patients were excluded if the diagnosis was uncertain or lacked core identifiers required to link records across the registry and charts, or if the available documentation precluded extraction of essential baseline staging information at any level (e.g., absent TNM components and no reliable M-status documentation).

#### 2.2.2. Data Sources and Variables

Data were abstracted into a structured dataset (Microsoft Excel, Microsoft Corp., Redmond, WA, USA) from the institutional oncology registry and electronic medical records. Variables included:

• Demographics: Age at diagnosis and sex.
• Disease parameters: TNM components (T, N, M) according to the AJCC/UICC 8th edition framework, as recorded in clinical documentation; M status was used to define metastatic disease at diagnosis.
• Treatment allocation: Surgery (any surgical intervention recorded during the initial management course) and non-surgical management.
• Laboratory markers (when available): Serum tumor markers (CEA, CA19-9), where marker values were treated as continuous covariates without defining clinical cut-offs.

#### 2.2.3. Primary Endpoint and Sensitivity Endpoint

The primary endpoint was metastatic disease at diagnosis (M1), defined among patients with M status explicitly documented as M0 or M1 (referred to as M-defined). Patients with Mx were excluded from M1-specific analyses, with denominators reported accordingly.

A pre-specified sensitivity endpoint was locally advanced or metastatic disease (LAM) in the TNM-complete subset, defined as:

LAM = M1 OR T4 OR N2–N3.

This endpoint was used to assess whether patterns observed with the primary endpoint were directionally consistent in cases with full TNM information.

#### 2.2.4. Staging Completeness and Phenotypes

To represent real-world variability in staging pathways, incomplete staging was defined a priori as the presence of Tx and/or Nx and/or Mx at baseline documentation. This definition was applied uniformly across all patients.

For phenotype-based analyses, patients with complete TNM data were categorized as:

• M0–non-LAM (non-metastatic and not locally advanced),
• M0–LAM (non-metastatic but locally advanced per LAM definition),
• M1 (metastatic disease).

This pragmatic phenotype structure was selected to align with common real-world decision branches and to support interpretable summaries of treatment allocation.

#### 2.2.5. Handling of Missing Data

Given the retrospective nature of the dataset and the heterogeneous completeness of some fields, analyses were performed using complete-case approaches per analysis, with transparent reporting of denominators:

• M1 analyses were restricted to M-defined cases (M0/M1).
• LAM sensitivity analyses were restricted to TNM-complete cases.

No statistical imputation was performed, as missingness was considered potentially informative in the context of diagnostic pathway heterogeneity.

### 2.3. Statistical Analysis

Data preprocessing was undertaken in Microsoft Excel. Statistical analyses were performed in IBM SPSS Statistics v26 (IBM Corp., Armonk, NY, USA). Continuous variables are reported as mean ± standard deviation (SD) when approximately normally distributed and as median (interquartile range, IQR) otherwise; categorical variables are reported as counts and percentages.

Group comparisons used Student’s *t*-test for approximately normally distributed continuous variables, Mann–Whitney U for non-normal continuous variables, and the χ^2^ test for categorical variables, as appropriate. For phenotype-level comparisons across more than two groups, χ^2^ tests were applied for categorical outcomes and Kruskal–Wallis tests for continuous outcomes when relevant.

Multivariable modeling was used to assess independent associations:

• Logistic regression for metastatic disease (M1): Outcome M1 (yes/no) among M-defined cases, with covariates including age, sex, and tumor markers when available. Tumor markers were entered as continuous variables using the transformation log10 (marker + 1) to mitigate skewness and preserve rank information without imposing cut-offs.
• Logistic regression for surgery (treatment allocation): Surgery (yes/no) modeled against metastatic status (M1) and/or LAM in the relevant subsets, adjusting for age and sex as core covariates.

All tests were two-tailed with α = 0.05. Effect sizes are reported as odds ratios (ORs) with 95% confidence intervals (CIs).

### 2.4. Ethics

The study received approval from the Ethics Committee of the University of Medicine and Pharmacy of Craiova (approval number 18593/13 April 2023) and was conducted in accordance with the principles of the Declaration of Helsinki.

## 3. Results

### 3.1. Cohort Characteristics and Completeness of Staging

A total of 419 patients with gastric cancer diagnosed between 2018 and 2021 were included in the analysis. The mean age at diagnosis was 66.9 ± 12.0 years (median 69 IQR 59–76), and 62.3% of patients were male. Overall, 218 patients (52.0%) underwent surgical treatment, whereas 201 (48.0%) were managed without surgery.

Incomplete staging at diagnosis—defined a priori as the presence of Tx and/or Nx and/or Mx—was identified in 154 patients (36.8%), underscoring real-world variability in staging completeness. Despite this, M status was available in 375 patients (89.5%), among whom metastatic disease (M1) was present in 195 cases (52.0%) (Table 1).

Age-stratified analyses were additionally performed using three clinically interpretable age groups (<65, 65–74, and ≥75 years). Incomplete staging occurred in 36.5% of patients younger than 65 years, 33.8% of those aged 65–74 years, and 39.8% of patients aged 75 years or older. Among patients with defined M status, the proportion of metastatic disease was similar in the <65 and 65–74 age groups (54.0% and 54.6%, respectively) and slightly lower in patients aged ≥75 years (46.6%). Surgical treatment was performed in 54.5% of patients younger than 65 years, 53.1% of those aged 65–74 years, and 44.4% of patients aged ≥75 years.

### 3.2. Distribution of T, N, and M Categories at Diagnosis

Across the cohort, the TNM components showed a predominance of advanced local and nodal disease, alongside a high burden of metastatic presentation. Tx and Nx entries were frequent, consistent with the substantial proportion of incomplete staging. The M component was clearly defined as M0 or M1 in 375 patients (89.5%). Full distributions are provided in Table 2.

A small number of records contained non-standard TNM entries (e.g., ‘GIST’, ‘LIMF’), reflecting real-world heterogeneity in clinical documentation; these were retained for descriptive reporting but excluded from models requiring standard M0/M1 classification.

### 3.3. Complete Versus Incomplete Staging at Diagnosis

Patients with incomplete staging (Tx and/or Nx and/or Mx) did not differ materially in age or sex distribution compared with completely staged patients; however, they were substantially less likely to undergo surgery, indicating that incomplete staging is clinically consequential rather than random missingness (Table 3).

### 3.4. Metastatic Versus Non-Metastatic Disease at Presentation

Among patients with documented M status (*n* = 375), metastatic disease (M1) was present in 195 cases (52.0%). Age and sex distributions were similar between M0 and M1 groups, whereas treatment allocation differed markedly: surgery was performed in 73.3% of M0 patients versus 34.4% of M1 patients (Table 4).

### 3.5. Clinical Phenotypes of Advanced Gastric Cancer

Among patients with complete TNM staging (*n* = 262), three clinically relevant phenotypes were identified: M0 non–locally advanced malignancy (M0 non-LAM), M0 locally advanced malignancy (M0 LAM), and metastatic disease (M1). M0 LAM represented the largest subgroup, accounting for 92 patients (35.1%), followed by M1 disease in 95 patients (36.3%) and M0 non-LAM in 75 patients (28.6%) (Table 5).

M0 non-LAM indicates non-metastatic, non–locally advanced disease; M0 LAM indicates non-metastatic locally advanced disease (T4 and/or N2–N3); M1 indicates metastatic disease at diagnosis. Percentages are calculated within each phenotype.

Marked differences in treatment allocation were observed across phenotypes. Patients with M0 LAM exhibited the highest likelihood of undergoing surgical treatment (89.1%), reflecting the predominance of potentially resectable but advanced local disease. In contrast, surgical intervention was less frequent among patients with M0 non-LAM (62.7%) and lowest among those with metastatic disease (48.4%), underscoring the impact of disease extent on therapeutic decision-making. Surgical allocation differed significantly across phenotypes (χ^2^ test, *p* < 0.001; Table 5).

**Figure 1:**
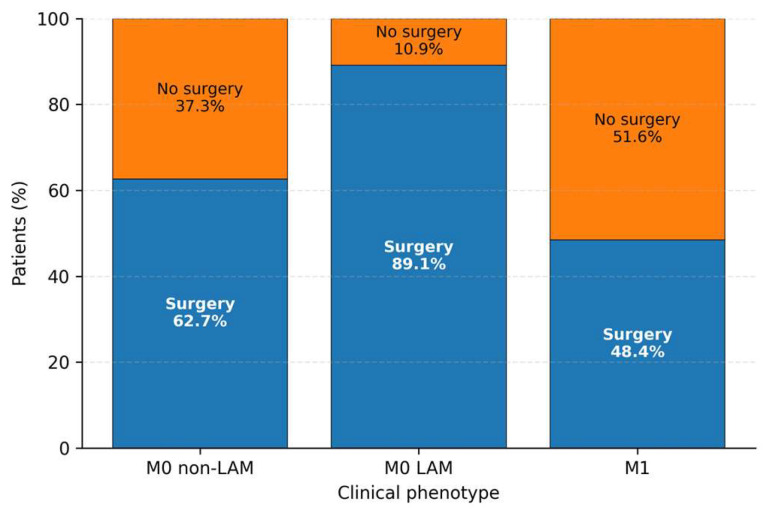
Surgical treatment rates across clinical phenotypes of gastric cancer. Bar plot showing the proportion of patients undergoing surgery according to clinical phenotype (M0 non-LAM, M0 LAM, and M1) among patients with complete TNM staging.

Median age differed modestly across phenotypes, with M0 non-LAM patients being slightly older than those with M0 LAM and M1 disease. These findings highlight the heterogeneity of advanced gastric cancer at diagnosis and demonstrate that phenotypic stratification based on TNM components captures clinically meaningful differences in management patterns within a real-world cohort (Figure 1).

### 3.6. Patterns and Clinical Correlates of Incomplete Staging

Incomplete staging at diagnosis—defined a priori as the presence of Tx, Nx, and/or Mx—was observed in 154 of 419 patients (36.8%), indicating substantial real-world variability in the completeness of initial diagnostic work-up. Among incompletely staged cases, Nx was the most frequent component, followed by Tx and Mx, whereas combined patterns involving more than one missing TNM component were less common (Table 6). Notably, the Tx-only subgroup had no recorded surgical intervention, consistent with limited operability and/or constrained treatment documentation in this pattern (Table 6).

Incomplete staging was clinically heterogeneous. Tx-only and Nx-only patterns were associated with very high proportions of metastatic disease among cases with subsequently documented M status, whereas the Tx + Nx subgroup showed intermediate metastatic burden. Notably, the Tx + Nx + Mx pattern reflected major constraints in staging completeness (including Mx), and was accompanied by low surgical utilization (Table 6).

When compared with patients with complete TNM staging, incompletely staged patients were significantly less likely to receive surgical treatment, despite similar age and sex distributions. This finding suggests that incomplete staging reflects not random missingness but rather constrained diagnostic pathways in clinically advanced or complex presentations, with direct implications for treatment allocation.

Taken together, these results indicate that incomplete staging represents a clinically meaningful marker of diagnostic trajectory in gastric cancer, closely linked to metastatic burden and downstream therapeutic decision-making rather than solely to data availability.

### 3.7. Multivariable Associations with Metastatic Disease and Incomplete Staging

In multivariable logistic regression adjusted for age (per 10 years), sex (male vs. female), and tumor markers (log10 (CEA + 1), log10 (CA19-9 + 1)), none of these covariates showed a statistically significant independent association with metastatic presentation (M1) at diagnosis, although CA19-9 showed a borderline association (*p* = 0.074) (Table 7). By contrast, metastatic disease was strongly associated with incomplete staging (Tx/Nx/Mx), even after adjustment (Table 8), suggesting that advanced presentation is linked to constrained or abbreviated staging workflows. These associations are reported to contextualize metastatic presentation and should not be interpreted as a diagnostic scoring tool.

### 3.8. Determinants of Surgical Treatment Allocation

In the adjusted model for surgical treatment, metastatic disease at diagnosis was the dominant determinant of non-surgical management (Table 9). Age and sex did not retain independent associations with surgery after accounting for M status.

### 3.9. Sensitivity Analyses Using the LAM Composite Endpoint

**Figure 2:**
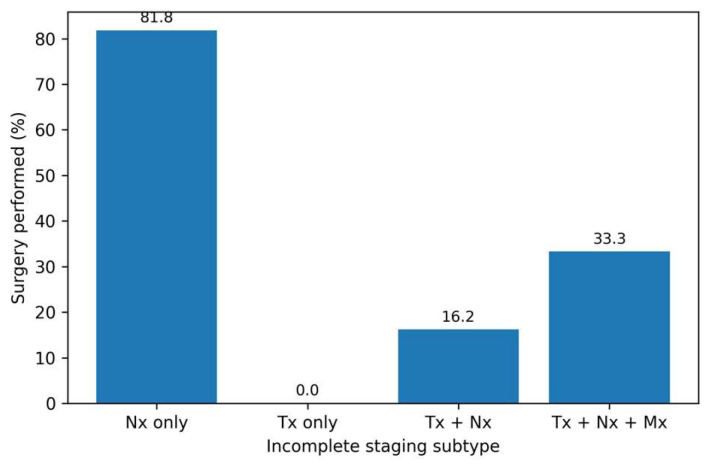
Comparison of surgical intervention rates across incomplete staging subtypes. Bar plot showing the proportion of surgery (yes/no) by incomplete staging subtype (Tx-only, Nx-only, Tx + Nx, Tx + Nx + Mx).

Sensitivity analyses using the composite endpoint of locally advanced or metastatic disease (LAM; M1 or T4 or N2–N3) in the TNM-complete subset yielded directionally consistent findings(Figure 2). In this subset, age and sex showed modest associations with LAM status (Table 10), whereas LAM itself was not independently associated with surgery after adjustment (Table 11).

## 4. Discussion

### 4.1. Summary of Principal Findings

We analyzed 419 consecutive patients diagnosed with gastric cancer and three observations stand out. First, incomplete TNM documentation was common. Using a prespecified definition, Tx and/or Nx and/or Mx occurred in more than one third of cases, which means that TNM detail cannot be taken for granted in retrospective hospital datasets. This is not unique to our center. Minicozzi et al., within the EUROCARE 5 framework [27], reported substantial heterogeneity in stage at diagnosis availability across European cancer registries and emphasized that incomplete or non-comparable stage data can materially change stage distributions and complicate between-cohort comparisons [28,29,30,31,32].

Second, metastatic disease at diagnosis was frequent among patients with a recorded M category. In our dataset, M status was available in most patients, and among those with defined M, roughly half were M1. As expected, M1 status coincided with substantially lower use of surgery at presentation, in line with guideline pathways where resection is generally pursued for non-metastatic disease and only selectively considered in metastatic settings [11,33]. Age-stratified analyses showed broadly similar patterns of metastatic presentation across age groups, although surgical treatment was somewhat less frequent among patients aged 75 years or older, reflecting the influence of age and clinical context on treatment allocation in real-world settings.

Third, the phenotype grouping used in TNM-complete cases, M0-nonLAM, M0-LAM, and M1, helped translate TNM detail into categories that resemble real clinical decision branches. It also provided a practical scaffold for sensitivity analyses restricted to patients with complete TNM information.

### 4.2. Incomplete Staging as a Signal of the Diagnostic Pathway

Rather than treating Tx, Nx, or Mx purely as a technical nuisance, we approached incomplete staging as a measurable feature of routine care. Registry methodology studies have repeatedly shown that TNM completeness varies by setting and over time, and that different assumptions about missing N or M can materially change downstream analyses, including survival inferences [34,35]. In our cohort, incomplete staging was too frequent to be dismissed as a rare artifact, so we reported it directly and incorporated it into sensitivity analyses.

Consistent with registry methodology literature, ‘unknown’ stage frequently reflects predictable documentation processes that depend on multi-source ascertainment, rather than exceptional events, and should therefore be reported explicitly in real-world cohorts [27,36].

A useful counterpoint is that incomplete TNM is not inevitable. McPhail et al. [37] reported that in England, stage completeness reached a level that supported robust, national stage-specific analyses, illustrating that systematic improvements in capture and linkage can substantially reduce the “unknown stage” problem. In other words, our findings are likely to reflect both disease burden and modifiable system factors. This strengthens the case for reporting TNM completeness explicitly and for interpreting retrospective cohorts with caution when complete staging is assumed by default.

### 4.3. Advanced Disease Phenotypes and Treatment Selection

The strong link between metastatic status and lower likelihood of surgery should be read as a treatment selection pattern in a retrospective cohort, not as a causal effect. Guidelines draw a clear management distinction between localized or locally advanced disease and metastatic disease, with systemic therapy forming the backbone of treatment in metastatic presentations and surgery reserved for selected situations.

Phenotype-based summaries may help bridge the gap between registry-friendly staging variables and the way decisions are made in clinic. In TNM-complete cases, surgery clustered in the non-metastatic locally advanced phenotype, while it was least frequent in M1. This pattern is coherent with how many centers triage patients at diagnosis. Comparative data from routine-care cohorts also reinforce the broader picture. Freile et al. [38] observed that stage IV disease accounted for roughly 44% of diagnoses in both European and Latin American hospital-based cohorts, which helps contextualize why treatment allocation is often constrained by advanced presentation. In parallel, Zhang et al., using SEER-based data focused on stage IV gastric cancer, described the metastatic burden and patterns of distant spread in a population-level setting, which reinforces that disseminated disease is not a rare phenotype at the time patients enter the system [39].

A reasonable counterargument is that tertiary center cohorts may overrepresent complex and advanced cases, which can inflate estimates of metastatic presentation compared with population-based registries. That possibility does not undermine the association between M1 and reduced surgery, but it does affect generalizability and should be acknowledged.

### 4.4. CEA and CA19-9 as Supportive, Not Determinative, Signals

CEA and CA19-9 are attractive in retrospective datasets because they are often available and easy to extract; however, the literature consistently frames them as adjunctive rather than decisive biomarkers. At the same time, contemporary gastric cancer management increasingly incorporates molecularly targeted strategies, particularly in HER2-positive disease, highlighting the broader shift toward biologically informed treatment selection in gastric cancer [40]. In our models, we treated markers as continuous covariates without imposing cut-offs. Both markers showed directionally plausible associations with metastatic presentation, but effect sizes were modest and uncertainty remained.

This cautious signal is compatible with prior work, although many studies take a different analytic route. Shibata et al. evaluated postoperative monitoring after curative gastrectomy and concluded that CA19-9 may be more useful than CEA for recurrence detection, an approach that relies on clinical thresholds and standardized follow-up rather than continuous modeling in heterogeneous diagnostic datasets [21].

Earlier studies such as Liu et al. also combined marker concentrations to stratify prognosis after curative resection, again typically using predefined cut-offs and more uniform measurement schedules [41].

Against that background, our results can be read as follows. Marker levels may carry biological context, but without prespecified thresholds and with non-uniform capture they should not be interpreted as stand-alone tools for staging or phenotype assignment. This framing also reduces the risk of overclaiming in a real-world cohort where laboratory testing is not standardized.

### 4.5. Strengths, Limitations, and Implications

A major strength of this study is that staging completeness was defined upfront and treated as part of the analytic problem, rather than being handled implicitly. The phenotype framework increased interpretability and allowed a structured sensitivity analysis in TNM-complete cases, which helped test whether observed patterns were robust to differences in documentation completeness.

Limitations reflect the retrospective design. Data completeness is variable, documentation practices can introduce bias, and unmeasured confounders can influence observed associations, especially around treatment allocation. Tumor marker analyses were further constrained by missingness, which reduced effective sample size and precision. These results should therefore be considered hypothesis-generating rather than definitive.

Future studies could extend this work by linking diagnostic phenotypes to longitudinal outcomes, improving harmonization of staging documentation, and validating the phenotype framework and marker associations in independent cohorts. Multi-center real-world initiatives in gastric cancer illustrate how harmonized data capture can improve comparability and strengthen inference.

## 5. Conclusions

This real-world analysis of patients with gastric cancer highlights substantial heterogeneity in diagnostic staging pathways and treatment allocation at presentation. Incomplete TNM staging—defined using a transparent and reproducible operational rule (Tx and/or Nx and/or Mx)—was frequent and should be explicitly acknowledged when interpreting retrospective oncology datasets derived from routine clinical practice.

Metastatic disease at diagnosis (M1) was common among patients with defined M status and was strongly associated with lower rates of surgical intervention, reflecting guideline-concordant treatment selection in advanced disease. Phenotype-based stratification integrating metastatic and locally advanced features provided a clinically interpretable framework that aligned with real-world decision branches and facilitated robust sensitivity analyses restricted to TNM-complete cases. Age-stratified analyses further suggest that diagnostic documentation and treatment allocation patterns should be interpreted within real-world clinical pathways, particularly in older patients who may follow more constrained diagnostic trajectories.

Serum tumour markers (CEA and CA19-9), analysed as continuous variables without predefined cut-offs, showed directionally coherent but non-definitive associations with metastatic presentation; therefore, in heterogeneous real-world datasets they should be interpreted as supportive contextual elements rather than determinative staging tools.

## Figures and Tables

**Table 1:** Baseline demographic and clinical characteristics of the study cohort.

Characteristic	Value
Patients, *n*	419
Age, years (mean ± SD)	66.9 ± 12.0
Age, years (median [IQR])	69 [59–76]
Sex, male, *n* (%)	261 (62.3%)
Sex, female, *n* (%)	158 (37.7%)
Surgery (any), yes, *n* (%)	218 (52.0%)
Surgery (any), no, *n* (%)	201 (48.0%)
Incomplete staging (Tx and/or Nx and/or Mx), *n* (%)	154 (36.8%)
M status defined (M0/M1), *n* (%)	375 (89.5%)
Metastatic disease among M-defined (M1), *n* (%)	195 (52.0%)

**Table 2:** Distribution of T, N, and M categories at diagnosis.

Domain	Level	*n*	%
T-stage	T3	139	33.2
	TX	132	31.5
	T4	115	27.4
	T2	27	6.4
	T1	4	1.0
	LIMF	1	0.2
	TIS	1	0.2
N-stage	NX	123	29.4
	N0	83	19.8
	N3	75	17.9
	N1	74	17.7
	N2	63	15.0
	LIMF	1	0.2
M category	M1	195	46.5
	M0	180	43.0
	MX	33	7.9
	GIST	7	1.7
	LIMF	4	1.0

Note: Percentages in Table 2 are calculated from the full cohort (*N* = 419). Percentages among M-defined cases are reported in Table 1.

**Table 3:** Comparison between patients with complete and incomplete staging at diagnosis.

Variable	Staging		*p*-Value
	Complete	Incomplete	
Age, years (median [IQR])	68 [60–76]	69 [58–77]	0.786 *
Male sex, *n* (%)	161 (60.8%)	100 (64.9%)	0.455 **
Surgery (any), *n* (%)	178 (67.2%)	40 (26.0%)	<0.001 **

* Mann–Whitney U test; ** Chi-square test. Values in bold indicate statistically significant differences (*p* < 0.05).

**Table 4:** Comparison of demographic and clinical characteristics between M0 and M1 patients.

Variable	M0	M1	*p*-Value
Age, years (median [IQR])	70 [61–77]	68 [58–76]	0.158 *
Male sex, *n* (%)	116 (64.4%)	115 (59.0%)	0.326 **
Surgery (any), *n* (%)	132 (73.3%)	67 (34.4%)	<0.001 **

* Mann–Whitney U test; ** Chi-square test. Values in bold indicate statistically significant differences (*p* < 0.05).

**Table 5:** Clinical phenotype groups based on TNM-complete cases and associated treatment allocation.

Phenotype	*n* (%)	Surgery Performed, *n* (%)	Median Age (Years)
M0 non-LAM	75 (28.6)	47 (62.7)	72
M0 LAM	92 (35.1)	82 (89.1)	67
M1	95 (36.3)	46 (48.4)	68

**Table 6:** Types of incomplete staging and associated clinical features.

Type of Incomplete Staging	*n* (%)	M1 Present, *n* (%)	Surgery Performed, *n* (%)
Nx only	22 (14.3)	20 (90.9)	18 (81.8)
Tx only	31 (20.1)	29 (93.5)	0 (0.0)
Tx + Nx	68 (44.2)	51 (75.0)	11 (16.2)
Tx + Nx + Mx	33 (21.4)	—	11 (33.3)
Total incomplete staging	154 (100)	—	—

Note: Tx, Nx, and Mx indicate unspecified primary tumor, nodal status, and metastatic status at diagnosis, respectively. Percentages for M1 disease and surgery are calculated within each incomplete staging subtype. For the Tx + Nx + Mx subtype, M status is undefined (Mx); therefore, the proportion of M1 is not applicable (—).

**Table 7:** Multivariable logistic regression for predictors of metastatic disease at diagnosis (M1). Model includes age, sex, and tumor markers (log10 (CEA + 1), log10 (CA19-9 + 1)).

Predictor	Adjusted OR	95% CI	*p*-Value
Intercept	2.96	0.88–9.93	0.079
Age (per 10 years)	1.00	0.82–1.34	0.823
Sex (male vs. female)	0.66	0.33–1.30	0.225
log10 (CEA + 1)	1.35	0.79–2.32	0.270
log10 (CA19-9 + 1)	1.44	0.97–2.14	0.074

Note: Sex is coded as male vs. female (female = reference category).

**Table 8:** Multivariable logistic regression for predictors of incomplete staging (Tx/Nx/Mx).

Predictor	Adjusted OR	95% CI		*p*-Value
		Low	High	
Intercept	0.03	0.01	0.15	**<0.001**
M1	15.41	8.00	29.65	**<0.001**
Age (per 10 years)	1.10	0.89	1.36	0.365
Sex (male vs. female)	1.34	0.80	2.27	0.269

Values in bold indicate statistically significant differences.

**Table 9:** Multivariable logistic regression for predictors of surgical treatment.

Predictor	Adjusted OR	95% CI		*p*-Value
		Low	High	
Intercept	6.68	1.72	25.99	**0.006**
M1	0.18	0.12	0.29	**<0.001**
Age (per 10 years)	0.87	0.72	1.05	0.137
Sex (male vs. female)	1.13	0.72	1.77	0.609

Values in bold indicate statistically significant differences.

**Table 10:** Sensitivity analysis: predictors of LAM (TNM-complete subset).

Predictor	Adjusted OR	95% CI		*p*-Value
		Low	High	
Intercept	23.16	3.58	149.68	**0.006**
Age (per 10 years)	0.77	0.60	1.00	**0.047**
Sex (male vs. female)	0.47	0.26	0.85	**0.012**

Values in bold indicate statistically significant differences. Note: Sex is coded as male vs. female (female = reference category).

**Table 11:** Sensitivity analysis: predictors of surgical treatment in the TNM-complete subset (including LAM).

Predictor	Adjusted OR	95% CI		*p*-Value
		Low	High	
Intercept	7.44	1.24	44.53	**0.028**
LAM	1.24	0.70	2.21	0.457
Age (per 10 years)	0.80	0.63	1.01	0.057
Sex (male vs. female)	1.13	0.66	1.94	0.662

Values in bold indicate statistically significant differences. Note: Sex is coded as male vs. female (female = reference category).

## Data Availability

The original contributions presented in this study are included in the article. Further inquiries can be directed to the corresponding authors.
